# Reduction of Tissue Na^+^ Accumulation After Renal Transplantation

**DOI:** 10.1016/j.ekir.2021.06.022

**Published:** 2021-06-28

**Authors:** Anke Dahlmann, Peter Linz, Isabelle Zucker, Viktor Haag, Jonathan Jantsch, Thomas Dienemann, Armin M. Nagel, Patrick Neubert, Daniela Rosenhauer, Manfred Rauh, Stephan Horn, Dominik N. Müller, Mario Schiffer, Friedrich C. Luft, Michael Uder, Christoph Kopp

**Affiliations:** 1 Department of Nephrology and Hypertension, Friedrich-Alexander-University Erlangen-Nürnberg, Erlangen, Germany; 2 Institute of Radiology, Friedrich-Alexander-University Erlangen-Nürnberg, Erlangen, Germany; 3 Institute for Medical Microbiology and Hygiene, University Regensburg, Regensburg, Germany; 4 Department of Surgery, University Regensburg, Regensburg, Germany; 5 Division of Medical Physics in Radiology, German Cancer Research Centre (DKFZ), Heidelberg, Germany; 6 Department of Pediatrics, Friedrich-Alexander-University Erlangen-Nürnberg, Erlangen, Germany; 7 Kuratorium für Heimdialyse, Erlangen, Germany; 8 Experimental and Clinical Research Center, a joint cooperation between the Charité Medical Faculty and the Max-Delbrück Center for Molecular Medicine, Berlin, Germany

**Keywords:** chronic kidney disease, kidney transplantation, tissue Na^+^, ^23^Na–magnetic resonance imaging, vascular endothelial growth factor C

## Abstract

**Introduction:**

Chronic kidney disease (CKD) engenders salt-sensitive hypertension. Whether or not tissue Na^+^ accumulation is increased in CKD patients remains uncertain. How tissue Na^+^ is affected after renal transplantation has not been assessed.

**Methods:**

We measured tissue Na^+^ amount in 31 CKD patients (stage 5) and prospectively evaluated tissue Na^+^ content at 3 and 6 months, following living-donor kidney transplantation. Additionally, pre- and post-transplantation data were compared to 31 age- and sex-matched control subjects. ^23^Na–magnetic resonance imaging (^23^Na-MRI) was used to quantify muscle and skin Na^+^ of the lower leg and water distribution was assessed by bioimpedance spectroscopy.

**Results:**

Compared to control subjects, CKD patients showed increased muscle (20.7 ± 5.0 vs. 15.5 ± 1.8 arbitrary units [a.u.], *P* < 0.001) and skin Na^+^ content (21.4 ± 7.7 vs. 15.0 ± 2.3 a.u., *P* < 0.001), whereas plasma Na^+^ concentration did not differ between groups. Restoration of kidney function by successful renal transplantation was accompanied by mobilization of tissue Na^+^ from muscle (20.7 ± 5.0 vs. 16.8 ± 2.8 a.u., *P* < 0.001) and skin tissue (21.4 ± 7.7 vs. 16.8 ± 5.2 a.u., *P* < 0.001). The reduction of tissue Na^+^ after transplantation was associated with improved renal function, normalization of blood pressure as well as an increase in lymphatic growth-factor concentration (vascular endothelial growth factor C [VEGF-C] 4.5 ± 1.8 vs. 6.7 ± 2.7 ng/ml, *P* < 0.01).

**Conclusions:**

Tissue Na^+^ accumulation in predialysis patients with CKD was almost completely reversed to the level of healthy controls after successful kidney transplantation.

## Graphical abstract

**Figure:**
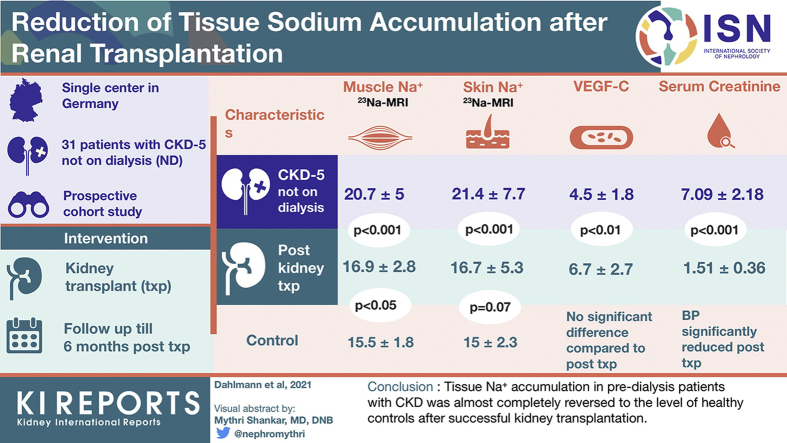

CKD is a major public health care burden as the prevalence is high, particularly within aging societies.^1^^,^^2^ Hypertension is one of the primary risk factors for cardiovascular disease, the main cause of morbidity and mortality in CKD patients.^3^ Salt (NaCl) intake is said to be largely responsible for hypertension in CKD patients and guidelines recommend salt restriction to ameliorate renal function loss and target-organ damage.^4^ Recent findings indicate that Na^+^ can accumulate and be stored at the tissue level bound to glycosaminoglycans.^5^^,^^6^ Tissue Na^+^ deposition might be a relevant mechanism of Na^+^-homeostasis alongside adjustments to extracellular fluid expansion in CKD patients. Previously, we introduced ^23^Na-MRI at our facility to visualize and quantify tissue Na^+^ noninvasively.^7^ We found that primary and secondary hypertension are accompanied by pronounced tissue Na^+^ retention, and that treatment of hyperaldosteronism is able to reverse this pathological condition.^8^ Furthermore, skin Na^+^ content in patients with mild to moderate CKD was the best predictor for left ventricular hypertrophy, indicating a close relationship between tissue Na^+^ deposition and target-organ damage.^9^ Tissue Na^+^ accumulation occurs in maintenance hemodialysis (HD) and peritoneal dialysis patients.^10^ HD patients with type 2 diabetes mellitus who are at a particularly high risk for cardiovascular disease have higher tissue Na^+^ content compared to nondiabetic HD patients.^11^ Removal of tissue Na^+^ in HD patients during a regular 4-hour dialysis treatment is independent of dialysis ultrafiltration, whereas the concentration of VEGF-C, a lymphatic growth factor, correlates with skin Na^+^ mobilization in these patients. VEGF-C is suppressed in HD patients while in parallel, soluble vascular endothelia growth factor receptor 3 (sVEGFR3) — a trap for VEGF-C — is increased.^12^ These findings are in line with previous preclinical studies showing that the lymphatic system plays an important role in tissue electrolyte clearance and blood pressure regulation.^6^^,^^13^, ^14^

In contrast to HD patients, data on tissue Na^+^ deposition in patients with severe CKD are limited and show conflicting results.^15^^,^^16^ Furthermore, whether the restoration of renal function by kidney transplantation is associated with a normalization of tissue Na^+^ amount is not known. We hypothesized that tissue Na^+^ accumulation occurring in predialysis CKD patients would be reduced within 6 months after successful living-donor kidney transplantation. In addition, we investigated potential underlying mechanisms of this tissue Na^+^ mobilization.

## Methods

### Study Population

The local ethics Committee of the University of Erlangen–Nürnberg, Germany (No. 3948 and 271_17B) approved the study, which was conducted according to the Declaration of Helsinki. All study participants provided written informed consent and were assessed at our clinical research facility at the University Hospital Erlangen. Patients were recruited from our transplantation center and our outpatient clinic. Patients with stable CKD stage 5 (estimated glomerular filtration rate < 15 ml/min per 1.73 m^2^) scheduled for pre-emptive living-donor transplantation were included in our prospective cohort study. None of the participants has been on dialysis treatment before the first ^23^Na-MRI assessment. Twenty-eight patients received pre-emptive living-donor transplantation; however, 3 patients required short-term HD treatment before transplantation. The study sample size was determined using G∗Power software for power analyses of *t* test.^17^ With 0.80 power, adjusted type I error test at level 5%, and an expected reduction of tissue Na^+^ of 5.2 ± 6.2 a.u. after intervention as found in our previous dialysis study, a minimum of 14 subjects per group were necessary.^12^ Exclusion criteria were patients with implanted metal, pregnant women, body mass index ≥ 40 kg/m^2^, malignant disease within the last 2 years, autoimmune disease, acute infection, surgery within the last 3 months, liver cirrhosis (Child C), congestive heart failure (New York Heart Association functional class IV), and patients meeting acute dialysis criteria. Diagnoses were obtained from medical reports. Patients were compared to age- and sex-matched healthy control subjects. Control subjects were recruited by newspaper advertisement. Recruitment of all study participants took place between January 2012 and November 2017. Healthy controls did not receive regular medication, except for L-thyroxine (two subjects), a contraceptive tablet (one subject), and glaucoma eye drops (one subject). Blood pressure of control subjects had to be within the normal range (<140/90 mm Hg) as defined by European Society of Hypertension/European Society of Cardiology. Participants underwent a blood and urine laboratory workup (including 24-hour urine samples) and were assessed for their medical history. Before MRI examination, blood pressure was measured after 5 minutes of rest in seated position using an automated oscillometric device (Dinamap, Critikon, Carlsbad, California, USA). Before recording, blood pressure was analyzed on both upper arms and the arm with higher pressure was chosen. Three consecutive measurements were averaged.

CKD patients were followed in a prospective cohort study. Follow-up examinations of tissue Na^+^ and clinical parameters were performed 3 and 6 months after transplantation and medical history after transplantation was assessed by questionnaire and medical reports. Follow-up ended on May 2018. The detailed immunosuppressive regime of the kidney transplant patients is depicted in Table 1.

**Table 1:** Clinical characteristics of chronic kidney disease patients pre– and 3 to 6 months post– renal transplantation and control subjects^a^

Characteristics	CKD	3 Months After Transplantation	6 Months After Transplantation	Controls
	(n = 31)	(n = 29)	(n = 31)	(n = 31)
Demographics				
Women/men, n	9/22	8/21	9/22	9/22
Age, y	48.0 ± 13.2	—	—	48.2 ± 13.6
eGFR (CKD-EPI), ml/min	9 ± 3	52 ± 12^d^	53 ± 14^d^	95 ± 13^b^^,^^c^
Body mass index, kg/m^2^	25.8 ± 3.8	25.7 ± 3.7	25.9 ± 3.8	24.1 ± 3.4
Body weight, kg	79.9 ± 18.1	79.9 ± 17.4	79.5 ± 18.0	75.4 ± 11.8
Systolic BP, mm Hg	140 ± 15	121 ± 14^d^	123 ± 13^d^	118 ± 12^b^
Diastolic BP, mmHg	87 ± 9	79 ± 9^d^	78 ± 9^d^	74 ± 8^b^
MAP, mm Hg	105 ± 10	92 ± 11^d^	93 ± 10^d^	89 ± 8^b^
Pulse pressure, mm Hg	53 ± 11	42 ± 8^d^	45 ± 11^d^	44 ± 10^b^
Comorbidities				
Hypertension	31	28	26	0
BP medication	3.0 ± 1.5	2.6 ± 1.2	2.0 ± 1.3^d^	*—*
Atrial fibrillation	1	2	2	0
Congestive heart failure	1	1	1	0
Coronary artery disease	0	0	0	0
Diabetes mellitus	2	2	2	0
Post-transplant diabetes	—	4	1	—
BIS data				
Total body water, liter	43.2 ± 9.1	40.5 ± 8.2^d^	40.7 ± 8.2^d^	40.6 ± 6.6
ECW, l	19.7 ± 4.4	18.5 ± 4.2^d^	18.5 ± 4.0^d^	17.7 ± 2.4^b^
ICW, l	23.5 ± 5.0	22.0 ± 4.2^d^	22.2 ± 4.4^d^	22.9 ± 4.2
Ratio ECW/ICW	0.84 ± 0.09	0.84 ± 0.07	0.84 ± 0.09	0.78 ± 0.06^b^^,^^c^
Biochemistry serum				
Potassium, mmol/l	4.7 ± 0.4	4.2 ± 0.5^d^	4.1 ± 0.4^d^	4.0 ± 0.3^b^
Creatinine, mg/dl	7.1 ± 2.2	1.5 ± 0.2^d^	1.5 ± 0.4^d^	0.9 ± 1.1^b^^,^^c^
hs CRP, mg/dl	3.3 ± 3.8	4.9 ± 14.5	7.4 ± 19.0	1.7 ± 2.0^b^
Glucose, mg/dl	104 ± 30	109 ± 15^d^	112 ± 24	98 ± 19^c^
HbA_1_c, %	5.3 ± 0.5	6.0 ± 0.8^d^	5.7 ± 0.5^d^	5.4 ± 0.3
Uric acid, mg/dl	7.4 ± 1.7	6.6 ± 1.7	6.8 ± 1.4	5.0 ± 1.2^b^^,^^c^
Lactate, mg/dl	0.9 ± 0.4	1.2 ± 0.4^d^	1.2 ± 0.4^d^	1.5 ± 1.3^b^
Triglyceride, mg/dl	198 ± 99	204 ± 83	199 ± 96	120 ± 77^b^^c^
Cholesterol, mg/dl	209 ± 46	204 ± 50	202 ± 55	203 ± 40
LDL, mg/dl	141 ± 38	131 ± 39	127 ± 40	125 ± 32^b^
Aldosterone, ng/ml	0.21 ± 0.16	0.08 ± 0.05^d^	0.10 ± 0.25^d^	0.10 ± 0.06^b^
Biochemistry urine				
Urine Na^+^, mmol/24 h	204 ± 86	195 ± 95	179 ± 82	187 ± 78
UACR, mg/g creatinine	2409 ± 1977	66 ± 73^d^	48 ± 59^d^	18 ± 26^b^
U-osmolality, mosm/kg	285 ± 53	319 ± 110	319 ± 111	459 ± 169^b^^,^^c^

BIS, bioimpedance spectroscopy; BP, blood pressure; CKD, chronic kidney disease; CKD-EPI, Chronic Kidney Disease Epidemiology Collaboration; ECW, extracellular water; eGFR, estimated glomerular filtration rate; HbA_1_c, glycated hemoglobin; hs CRP, high-sensitivity C-reactive protein; ICW, intracellular water; LDL, low-density lipoprotein; MAP, mean arterial pressure; RT, renal transplantation; UACR, urine albumin-to-creatinine ratio.

a Variables are presented as mean ± standard deviation.

b *P*_(Control vs. CKD)_ < 0.05.

c *P*_(Control vs. RT 6 mo)_ < 0.05.

d *P*_(RT vs. CKD)_ < 0.05.

### Bioimpedance Spectroscopy

To determine the fluid status of the study participants, a bioimpedance device was used (Body Composition Monitor, Fresenius Medical Care, Bad Homburg, Germany). Electrodes were attached to the hand and the foot of the ipsilateral side of the patient and impedance spectroscopy was measured with frequencies reaching from 5 kHz to 1 MHz. While high frequencies pass through the whole-body water, very low frequencies are not able to penetrate cell membranes; thus, they only pass through the extracellular water (ECW) space. The generated impedance data are applied to calculate total body water, intracellular water, and ECW using the model proposed by Chamney *et al.*^18^ Additionally, the individual excess in ECW (overhydration), correcting for sex, age, and body mass was calculated.

### Tissue Na^+^ assessment by ^23^Na-MRI

We applied ^23^Na-MRI to quantify tissue Na^+^ in human skin and muscle as described previously.^8^^,^^12^ We measured tissue Na^+^ content in the region of the dorsal lower leg (at the largest circumference) with a ^23^Na volume coil (Stark-Contrast, Erlangen, Germany) and a 3.0 Tesla magnetic resonance imaging scanner (Magnetom Verio or Magnetom Skyra, Siemens Healthcare, Erlangen, Germany). For analysis, the left lower leg was placed on a cylindrical-shaped surface to avoid deviation in the Z-axis. We used a two-dimensional fast low angle shot sequence (total acquisition time =13.7 minutes, echo time = 2.07 ms, repetition time = 100 ms, flip angle = 90°, 128 averages, resolution: 3 × 3 × 30 mm^3^). Four tubes containing aqueous solutions with 10, 20, 30, and 40 mmol/l NaCl served as calibration standards by relating intensity to a concentration in a linear trend analysis. The low in-plane resolution results in partial volume effects; thus, giving an underestimation of the Na^+^ skin content because the skin thickness is approximately 1 mm. In addition, the Na^+^ concentration of subcutaneous fat tissue influences the measured skin Na^+^, and the fast decay of the ^23^Na-MRI signal can result in an underestimation of tissue Na^+^ amount. To account for these limitations, we labeled the tissue Na^+^ concentration obtained by the calibration as arbitrary units (a.u.). For the entire study, the compartments of the lower leg, namely, triceps surae muscle and cutis were identified by the same experienced physicist. The anatomical regions of interest were drawn guided by the anatomical image (T1-weighted fast low angle shot sequence). The skin of the dorsal lower leg located directly above the cylindrical-shaped surface of the calibration tube holder was used for Na^+^ assessment. In the Na^+^ image, a threshold of twice the background noise defined the border for pixels that were included in skin Na^+^ calculation. Examples of our Na^+^ image analysis can be found in a previous publication.^19^

### Statistical Analysis

We used the SPSS software (version 24.0, IBM, Armonk, New York, USA). Original output data and Syntax files used for analysis will be provided on request. *P* < 0.05 was considered significant and two-sided tests of hypotheses were used throughout. Group differences between patients and controls were tested using the Student *t* test with adjustment for unequal variance as needed, or the Wilcoxon rank sum test. Intraindividual differences of transplant recipients were tested with the paired *t* test or the Wilcoxon signed rank test where appropriate. Differences in proportions were assessed using the chi square test. Clinical, laboratory, and metabolic parameters were used in a univariate analysis to study associations with skin Na^+^ and muscle Na^+^. Because there were three measurements over time, a generalized estimating equation was used. Generalized estimating equation adjusts for intrasubject correlation and facilitates implementation of the Markov correlation structure, which is appropriate for modeling associations among measurements that are unequally spaced in time.

## Results

In the present study, 31 patients with CKD stage 5 underwent living-donor kidney transplantation and were prospectively assessed prior, and at 3 and 6 months after surgery (on average 3.1 ± 0.5 and 6.2 ± 0.4 months) to detect changes in tissue Na^+^ content after restored kidney function. Additionally, CKD patients were compared to 31 age- and sex-matched healthy control subjects. In our cohort, 28 patients received a pre-emptive transplantation and 3 patients required short-term HD before transplantation. None of the patients has been on dialysis treatment before the first ^23^Na-MRI measurement. Anthropometric data, comorbidities, and biochemistry including metabolic parameters of study participants are outlined in Table 2. Specific medication and underlying renal diseases are presented in Table 1. After renal transplantation, serum creatinine levels decreased in CKD patients from 7.09 ± 2.18 to 1.51 ± 0.24 mg/dl after 3 months, and were 1.51 ± 0.36 mg/dl after 6 months, whereas estimated glomerular filtration rate increased accordingly. No graft loss occurred. Blood pressure values as well as burden of antihypertensive medication were significantly reduced after transplantation.

**Table 2:** Underlying nephropathy and medication list of all 31 CKD patients pre– and 3 to 6 months post–renal transplantation

	CKD	3 months after transplantation	6 months after transplantation
Primary/underlying renal disease			
Hypertensive nephropathy	4	—	—
Diabetic nephropathy	1	—	—
ADPKD	8	—	—
Primary FSGS	4	—	—
IgA nephritis	7	—	—
Reflux nephropathy	4	—	—
Mesangioproliferative GN	1	—	—
Unknown	2	—	—
Medication			
Immunosuppression	1	29	31
Tacrolimus	0	25	27
Cyclosporine	0	3	1
Mycophenolate mofetil	0	29	30
Belatacept	0	1	1
Sirolimus	0	0	2
Azathioprine	0	0	1
Prednisolone	1	29	31
Daily dosage	1 mg/d	7.5 mg/d (IQR 2.5)	5 mg/d (IQR 0)
Antihypertensive medication	31	28	26
ARB	13	16	10
ACEi	6	7	8
Diuretics	14	2	3
Calcium antagonist	19	16	10
ß-Blocker	14	17	13
Aldosterone antagonist	0	0	1
Others	15	9	7
Bicarbonate	24	4	0
Statins	15	29	27
Phosphate binder	17	0	0
Antidiabetic medication (insulin, glinides, gliptins)	2	6	3

ADPKD, autosomal dominant polycystic kidney disease; ACEi, angiotensin-converting enzyme inhibitor; ARB, angiotensin receptor blocker; CKD, chronic kidney disease; FSGS, focal segmental glomerulosclerosis; GN, glomerulonephritis; IgA, immunoglobulin A; IQR, interquartile range.

**Figure 1:**
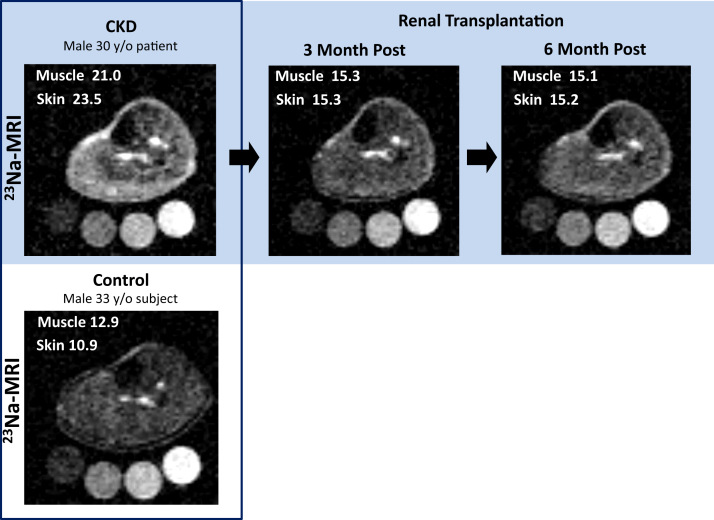
Representative ^23^Na–magnetic resonance images (^23^Na-MRIs) of a chronic kidney disease (CKD) patient (pre– and post–renal transplantation), and its age-matched control person. Upper panel shows ^23^Na-MRI of the left lower leg of a 30-year-old man with CKD stage 5 before (left image) as well as 3 months (middle image) and 6 months (right image) after pre-emptive living-donor kidney transplantation. Calibration tubes with 10, 20, 30, and 40 mmol/l are placed below the left lower leg. The brightness of the Na^+^ resonance signal in muscle and skin tissue is markedly reduced following renal transplantation — reaching signal intensities close to the ^23^Na-MRI of a 33-year-old healthy control man (see boxed images, lower panel).

**Figure 2:**
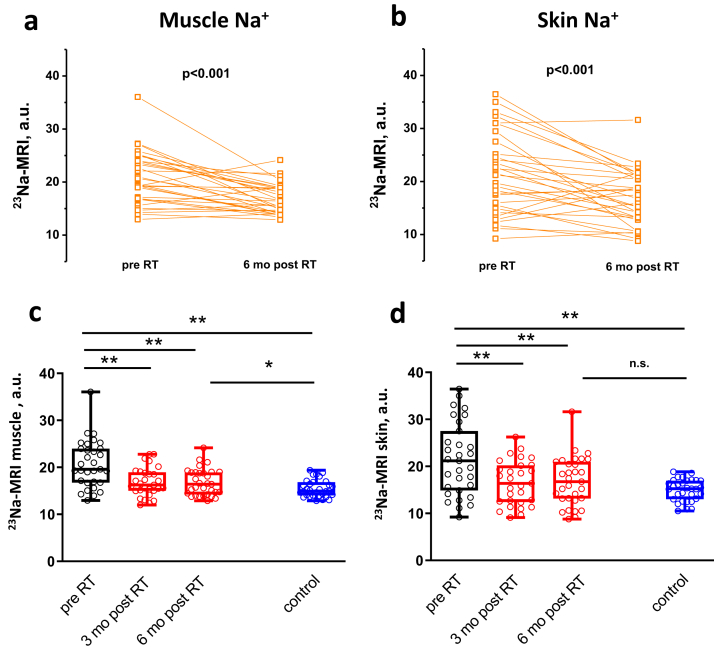
Changes in tissue Na^+^ content after renal transplantation (RT) and comparison to age- and sex-matched control subjects. Individual muscle **(a)** and skin **(b)** tissue Na^+^ significantly decreased after renal transplantation (n = 31). **(c)** Mean muscle Na^+^ content significantly decreased 3 months after transplantation and persisted on this level (red box plots, n = 29 [3 months], n = 31 [6 months]). Six months after surgery, muscle Na^+^ of kidney transplant recipients was still significantly higher compared to control subjects (blue box plot, n = 31)). **(d)** Mean skin Na^+^ content significantly decreased 3 months after transplantation and persisted on this level (red box plots, n = 29 [3 months], n = 31 [6 months]). Six months after surgery, skin Na^+^ of kidney transplant recipients was no longer significantly different compared to control subjects (blue box plot, n = 31). ∗∗*P* < 0.001. ∗*P* < 0.05. a.u., arbitrary units; MRI, magnetic resonance imaging; n.s. not significantly different.

Representative ^23^Na-MRIs of a CKD patient pre-surgery, 3 and 6 months post-surgery as well as a corresponding healthy control subject are depicted in Figure 1, indicating decreased tissue Na^+^ signaling after transplantation. Paired data from all 31 CKD patients show significant reduction of tissue Na^+^ content in muscle and skin after renal replacement therapy (Figure 2a and b). We note that the actual tissue Na^+^ content is underestimated by the methodology (see Methods section). Detailed assessment revealed a significant reduction of mean tissue Na^+^ content already 3 months after transplantation (20.7 ± 5.0 vs. 16.8 ± 3.0 a.u., *P* < 0.001 muscle and 21.4 ± 7.7 vs. 16.5 ± 4.7 a.u., *P* < 0.001 skin), which persisted on follow-up examination after 6 months (16.9 ± 2.8 a.u., *P* < 0.001, muscle and 16.7 ± 5.3 a.u., *P* < 0.001, skin) (Figure 2c and d, individual tissue Na^+^ data are depicted in Supplemental Table S1). Compared to age- and sex-matched healthy controls tissue, Na^+^ was significantly higher in CKD patients pretransplantation (skin 21.4 ± 7.7 vs. 15.0 ± 2.3 a.u., *P* < 0.001, muscle 20.7 ± 5.0 vs. 15.5 ± 1.8 a.u., *P* < 0.001). Despite the reduction, tissue Na^+^ did not reach the level of control subjects 6 months after transplantation, with a significant difference in muscle (15.5 ± 1.8 vs. 16.8 ± 2.8 a.u., *P* < 0.05) (Figure 2c), and only a tendency in skin (skin 15.0 ± 2.3 vs. 16.8 ± 5.2 a.u., *P* = 0.07) (Figure 2d).

In our cohort, six acute allograft rejections were detected histologically without a significant increase in serum creatinine compared to patients not experiencing rejection (no rejection 1.48 ± 0.27 vs. rejection 1.65 ± 0.65 mg/dl, *P* = 0.55 after 6 months). No difference in tissue Na^+^ amount could be detected in patients with acute rejection compared to patients without (skin 15.6 ± 5.1 vs. 17.1 ± 5.3 a.u., *P* = 0.52; muscle 17.0 ± 3.2 vs. 16.7 ± 2.8 a.u., *P* = 0.86) (Supplemental Table S2). Further subanalysis of CKD patients with diabetes mellitus (two patients with type 2 diabetes mellitus plus four patients with post-transplantation diabetes) as well as three CKD patients requiring short-term HD before surgery are also shown in Supplemental Table S2. No differences in tissue Na^+^ between diabetes and nondiabetes patients were observed.

**Figure 3:**
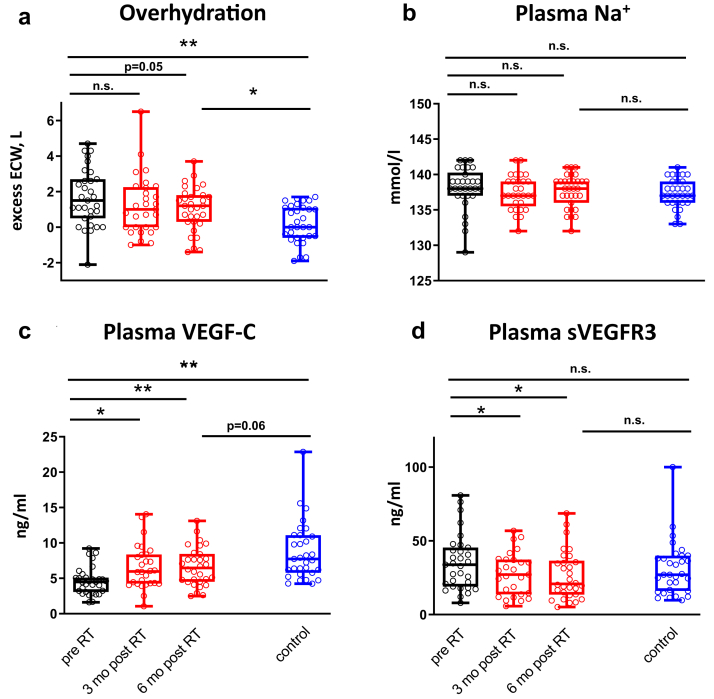
Changes in overhydration, plasma Na^+^, and lymphangiogenic factors after renal transplantation (RT) and comparison to age and sex-matched control subjects. **(a)** Overhydration did not significantly decrease after 3 months but was reduced 6 months after RT, albeit not reaching statistical significance (red box plots, n = 29 [3 months], n = 31 [6 months]). In kidney transplant recipients, overhydration remained significantly higher 6 months after surgery compared to control subjects (blue box plot, n = 31). **(b)** Plasma Na^+^ concentration was unaffected by RT (red box plots, n = 29 [3 months], n = 31 [6 months]) and did not differ 6 months after surgery between kidney transplant recipients and control subjects (blue box plot, n = 31). **(c)** Plasma vascular endothelial growth factor C (VEGF-C) concentration significantly increased 3 months after transplantation and persisted on this level (red box plots, n = 31 [chronic kidney disease (CKD)], n = 28 [3 months], n = 29 [6 months]). Six months after surgery a nonsignificant (n.s.) trend to lower plasma VEGF-C concentrations could be found in kidney transplant recipients compared to control subjects (blue box plot, n = 31). **(d)** Plasma soluble vascular endothelial growth factor receptor 3 (sVEGFR3) significantly decreased 3 months after transplantation and persisted on this level (red box plots, n = 31 (CKD), n = 28 [3 months], n = 29 [6 months]). Six months after surgery sVEGFR3 concentrations of kidney transplant recipients were not different compared to control subjects (blue box plot, n= 31). ∗∗*P* < 0.001. ∗*P* < 0.05. ECW, extracellular water.

There was a numerical reduction in overhydration assessed by bioimpedance spectroscopy (BIS) over the follow-up period; however, the significance level was not reached (1.7 ± 1.6 vs. 1.2 ± 1.7 l, *P* = 0.22 after 3 months and vs. 1.0 ± 1.2 l, *P* = 0.05 after 6 months) (Figure 3a). Additionally, BIS detected excess ECW (overhydration) in CKD patients (0.2 ± 1.1 vs. 1.7 ± 1.6 L, *P* < 0.001) (Figure 3a), whereas total body water was not significantly increased compared to controls, as shown in Table 2. No differences were observed in plasma Na^+^ concentration between control subjects and CKD patients (137 ± 2 vs. 138 ± 3 mmol/l, *P* = 0.27) (Figure 3b) and 24-hour urine Na^+^ amount (187 ± 78 vs. 204 ± 86 mmol/24 h, *P* = 0.46) (Table 2). The observed differences in tissue Na^+^ were paralleled by a persistent volume expansion in patients after transplantation compared to healthy controls (0.2 ± 1.1 vs. 1.0 ± 1.2 L, *P* < 0.01) (Figure 3a).

As the lymphatic system played a pivotal role in in tissue Na^+^ homeostasis in previous studies, we assessed lymphatic factors (VEGF-C and sVEGFR3) in the blood of our CKD patients. Following 3 and 6 months after transplantation, VEGF-C concentration significantly increased and sVEGFR3 decreased (VEGF-C: 4.5 ± 1.8 vs. 6.6 ± 3.1 (3 months) vs. 6.6 ± 2.7 ng/ml (6 months), *P* < 0.01; sVEGFR3: 34.4 ± 18.5 vs. 27.7 ± 14.5 (3 months) vs. 26.7 ± 16.5 ng/ml (6 months), *P* < 0.05) (Figure 3c and d). Compared to age- and sex-matched control subjects, VEGF-C concentrations in CKD patients were significantly lower and, although increasing, did not reach values of healthy control subjects 6 months after kidney transplantation. No significant differences in sVEGFR3 concentrations between CKD patients and control subjects could be detected (Figure 3d).

To assess factors that might influence changes in tissue Na^+^ after transplantation over time (including all three study visits), we investigated correlations between clinical, laboratory, and metabolic parameters by using a generalized estimating equation. We found that age, systolic blood pressure, creatinine, and overhydration were associated with muscle Na^+^ accumulation. The same correlations plus an association with gender and VEGF-C could be found for skin Na^+^ content, as shown in Table 3.

**Table 3:** Correlation between muscle Na^+^ or skin Na^+^ and various anthropometric, metabolic and lymphangiogenic parameters including all three study visits, assessed by the generalized estimating equation

Parameters	Univariate Variance analysis		
	Coefficient	*P*	95% Confidence Interval
Muscle Na^+^			
Age (per decade)	0.151	0.000	0.090 to 0.213
Sex	-1.379	0.232	-3.640 to 0.882
Systolic blood pressure	0.106	0.000	0.062 to 0.149
Overhydration	1.268	0.000	0.752 to 1.783
Creatinine	0.673	0.000	0.475 to 0.871
VEGF-C	-0.278	0.086	-0.595 to 0.039
sVEGFR3	0.029	0.311	-0.028 to 0.086
hs CRP	0.029	0.357	-0.033 to 0.091
HbA1c	-0.606	0.390	-1.988 to 0.775
LDL-cholesterol	0.022	0.051	-0.000 to 0.043
Aldosterone	0.286	0.905	-4.396 to 4.968
Skin Na^+^			
Age (per decade)	0.229	0.000	0.113 to 0.345
Sex	-6.036	0.001	-9.452 to -2.620
Systolic blood pressure	0.150	0.000	0.092 to 0.207
Overhydration	2.092	0.000	1.402 to 2.783
Creatinine	0.844	0.000	0.584 to 1.104
VEGF-C	-0.785	0.000	-1.193 to -0.378
sVEGFR3	-0.060	0.157	-0.144 to 0.023
hs CRP	-0.025	0.564	-0.110 to 0.060
HbA1c	-0.483	0.638	-2.496 to 1.531
LDL-cholesterol	0.016	0.324	-0.015 to 0.047
Aldosterone	1.219	0.704	-5.076 to 7.514

hs CRP, high sensitivity C-reactive protein; LDL, low-density lipoprotein; VEGF-C, vascular endothelia growth factor C; sVEGFR 3, soluble vascular endothelia growth factor receptor 3.

## Discussion

To date, transplantation is the best available renal replacement therapy in end-stage renal disease due to a higher survival rate of kidney transplant recipients compared to patients on maintenance dialysis.^20^ Whether this therapy is associated with a change in tissue Na^+^ content in CKD patients has not been evaluated before now. The main novel finding of our study was that tissue Na^+^ amount in CKD patients decreased after transplantation in muscle and skin tissue. Besides this, we were able to show that patients with pre–terminal CKD develop tissue Na^+^ overload compared to age- and sex-matched healthy control subjects. Tissue Na^+^ of CKD patients has been investigated by others. However, previous studies involving ^23^Na-MRI found either no difference in tissue Na^+^ content between CKD patients and healthy controls or solely in the subcutaneous compartment. The discrepancy to our data might be due to a wide variety of CKD stages and low patient numbers in the former and a relatively small control population in the later study.^15^^,^^16^ Assessment of skin biopsy specimens from renal transplant donors and recipients — pre-emptive and receiving HD — did not detect significant increased Na^+^ content.^21^ However, the examined HD patients were assessed directly after dialysis treatment, which might have reduced tissue Na^+^ according to our ^23^Na-MRI study in dialysis patients.^12^

In our investigation, a pronounced Na^+^ reduction in muscle and skin tissue could already be detected 3 months after surgery without any further significant changes after 6 months, indicating a rapid and persistent effect on tissue Na^+^ amount after renal transplantation. When compared to age-and sex-matched controls, Na^+^ was not completely normalized in muscle tissue post-transplantation, although all 31 patients obtained a stable kidney function throughout the end of our observational period (estimated glomerular filtration rate = 53 ± 14 ml/min per 1.73 m^2^) (Table 2). As we performed a prospective observational study and no randomized control trial, we were not able to prove a causative relationship between transplantation and tissue Na^+^ reduction. Multiple confounders might have additionally influenced tissue Na^+^ amount. Particularly, initiation of immunosuppressive therapy could have affected Na^+^/water distribution. Calcineurin inhibitors, which were administered to the majority (90%) of our kidney transplant recipients, are known to affect Na^+^ reuptake of the kidney by activating the Na^+^-Cl^-^-cotransporter.^22^ Glucocorticoid excess is able to stimulate renal Na^+^ reabsorption,^23^ whereas increased renal Na^+^ excretion was paralleled by high endogenous glucocorticoid concentrations in a human Na^+^ balance study.^24^ However, despite a significant reduction of glucocorticoid dosage from month 3 to month 6 post-transplantation (7.5 mg, interquartile range [IQR] 2.5 vs. 5 mg, IQR 0; *P* < 0.05) (Table 1), no equivalent changes in tissue Na^+^ occurred. This state-of-affairs indicates no direct correlation between tissue Na^+^ content and glucocorticoid intake in our study. Besides the immunosuppressant therapy, age, sex, and pre-existing diseases might all have interfered with tissue Na^+^ content and mobilization capacity following transplantation. Previous ^23^Na-MRI investigations revealed a sex-dependence and age-correlation of tissue Na^+^ storage.^7^^,^^10^ We have also found these correlations in our cohort pre- and post-transplantation. Furthermore, 2 of 31 CKD patients were diagnosed with type 2 diabetes mellitus, a condition known to enhance tissue Na^+^ accumulation in HD patients.^11^ Four patients additionally developed post-transplantation diabetes mellitus. A subgroup analysis of these six patients showed no difference in tissue Na^+^ amount after transplantation compared to CKD patients without diabetes (Supplemental Table S2). To determine further factors that might be relevant for the observed changes in tissue Na^+^ amount following transplantation, we assessed univariate regression models of tissue Na^+^ and clinical as well as laboratory parameters. Besides the above-mentioned parameters age and sex, excess ECW (overhydration), blood pressure, and VEGF-C showed a close association with tissue Na^+^ (Table 3). Because of the small number of patients, we were unable to perform a multivariate analysis to identify effects of different covariates on Na^+^ reduction.

We focused on CKD patients who were scheduled for pre-emptive living-donor kidney transplantation to avoid interferences of Na^+^ and water homeostasis by dialysis treatment as long-term chronic dialysis treatment is associated with various skin pathologies and thus might affect skin matrix and Na^+^ deposition.^25^ Skin biopsy results identified marked differences in collagen deposition, glycosaminoglycans, and lymph-angiogenesis between pre-emptive kidney transplant recipients and chronic dialysis patients.^21^ None of 31 CKD patients was receiving renal replacement therapy before the initial ^23^Na-MRI measurement. Although 28 patients were not dialyzed before kidney transplantation, 3 patients required short-term dialysis treatment before transplantation. These few patients revealed a similar pattern of tissue Na^+^ reduction after transplantation (Supplemental Table S2).

Although tissue Na^+^ was markedly mobilized 3 and 6 months after renal transplantation, excess extracellular water (overhydration) as assessed by BIS was not reduced 3 months after surgery. Six months after transplantation, a decrease of the initially detected overhydration was apparent, but not reaching level of significance. BIS has been widely used in studies on dialysis patients to determine fluid status more objectively. Even moderate fluid overload was thereby associated with increased cardiovascular mortality.^26^^,^^27^ In a cohort of 338 CKD patients not on dialysis treatment, hypervolemia was present in more than half of the study participants, which is comparable to the fluid status of our CKD cohort.^28^ In line with our data, a BIS trial investigating fluid status before and after transplantation revealed a high percentage of persistent overhydration 3 months after intervention.^29^ However, a recent study involving BIS and ^23^Na-MRI in CKD patients found that BIS measurements seem to overestimate water amount in study participants due to relying predominantly on tissue Na^+^ concentration and its conductance.^30^ This would be particularly relevant in light of tissue Na^+^ deposition, as BIS might rather reflect Na^+^ load than water amount and should be kept in mind when interpreting BIS-derived body water analysis in our CKD patients.

As previous basic-research and patient-oriented studies identified the lymphatic system as a considerable regulator of tissue Na^+^, we further characterized changes of the lymphangiogenic factors in our study participants. In animal experiments, NaCl-induced tissue hypertonicity resulted in macrophage-derived VEGF-C secretion and skin electrolyte drainage. Disruption of this adaption process by trapping of VEGF-C with sVEGFR3 leads to hypertonic volume retention in skin and elevated blood pressure.^6^^,^^13^, ^14^ We found that an initially reduced VEGF-C concentration in our CKD patients increased after transplantation, whereas sVEGFR3 concentration decreased. This altered pattern in lymphangiogenic factors has already been described in HD patients, where it was associated with reduced tissue Na^+^ mobilization capacity during a regular 4-hour HD treatment.^12^ VEGF-C differences between CKD patients and healthy subjects comparable to our study have been reported previously,^31^ whereas to our knowledge we are the first to describe changes of these lymphangiogenic factors after transplantation. However, the association between tissue Na^+^ and VEGF-C that occurred in our study does not prove causality. The observed changes might be due to multiple other factors such as therapeutic immunosuppressants or change of inflammation status. Furthermore, the kidney itself might be a source of lymphatic growth factors as in renal ischemia reperfusion models VEGF is transiently reduced until renal function recovers.^32^ In contrast to the above-mentioned animal experiments, no histological assessment of lymph capillary density and hyperplasia were performed in this study. Therefore, we do not know the direct impact of CKD and subsequent renal transplantation on the lymphatic network.

We are aware of general limitations of our study. As we did not collect data concerning dietary habits during our study, we do not know its impact on tissue Na^+^ content pre- and post-transplantation. However, the analysis of 24-hour urine Na^+^ excretion did not reveal differences over 3 time points within the CKD cohort nor in comparison to the control group. Besides the mentioned immunosuppressants, medication fundamentally changed after transplantation thereby possibly influencing Na^+^ homeostasis. At least no diuretics or sodium-glucose transport protein 2 inhibitors — both drugs known to induce tissue Na^+^ loss — were newly prescribed post transplantation (Table 1).^33^^,^^34^ A regression to the mean cannot be excluded in our data set. Finally, solely Caucasians were investigated in our study. Thus, results should not be generalized to all ethnicities, which is important as for instance African Americans are known to accumulate higher amounts of tissue Na^+^.^10^

The pathophysiological implications of tissue Na^+^ storage must be further elucidated. Recent data suggest an association between tissue Na^+^ accumulation and cardiovascular risk. In CKD patients, skin Na^+^ assessed by ^23^Na-MRI was the best predictive factor for left ventricular hypertrophy, a risk factor for cardiovascular morbidity and mortality.^9^ Primary aldosteronism, which causes myocardial damage independent of blood pressure, was found to be related not only to muscle and skin tissue Na^+^ accumulation but also to increased myocardial Na^+^ content.^35^ Prospective long-term cohort studies must reveal whether tissue Na^+^ overload *per se* is an independent risk factor for cardiovascular morbidity and mortality.

In summary, we present evidence that tissue Na^+^ accumulation occurs in CKD patients and that this overload was reduced after renal transplantation almost to the level of healthy controls. CKD was associated with alterations in lymphangiogenic plasma factors that were normalized after transplantation, indicating a role of lymphatic Na^+^ regulation in CKD. The ultimate relevance of Na^+^ accumulation for cardiovascular disease after transplantation and the role of allograft rejection with subsequent tissue Na^+^ retention should be addressed in further prospective long-term clinical trials.

## Disclosure

The authors declared no competing interests.

## Supplementary Material

Supplementary File (Word)
