# Supplementary material for: Reduction of Tissue Na+ Accumulation After Renal Transplantation
Source: Kidney Int Rep. 2021 Jun 28;6(9):2338–47. doi: 10.1016/j.ekir.2021.06.022 (PMC8418983; doi:10.1016/j.ekir.2021.06.022)
Supplement: Supplementary File (Word) [file mmc1.docx]

**Supplementary Table S1**

|  | **Na^+^ Muscle (a.u.)** | | | **Na^+^ Skin (a.u.)** | | |
| --- | --- | --- | --- | --- | --- | --- |
|  | **CKD** | **3 months** | **6 months** | **CKD** | **3 months** | **6 months** |
| 1 | 19.3 | 21.9 | 24.2 | 17.5 | 14.5 | 18.6 |
| 2 | 23.6 | 19.1 | 18.8 | 31.0 | 26.2 | 31.6 |
| 3 | 23.3 | 15.0 | 19.0 | 27.5 | 12.6 | 14.1 |
| 4 | 21.0 | 15.3 | 15.1 | 23.5 | 15.3 | 15.2 |
| 5 | 15.6 | **-** | 19.0 | 12.4 | - | 15.9 |
| 6 | 22.1 | 18.5 | 21.6 | 17.8 | 18.8 | 18.4 |
| 7 | 16.8 | 15.6 | 13.4 | 25.1 | 22.8 | 22.8 |
| 8 | 25.2 | 18.8 | 17.5 | 35.0 | 20.3 | 21.5 |
| 9 | 25.8 | 22.7 | 19.3 | 19.7 | 18.5 | 14.2 |
| 10 | 24.0 | **-** | 21.1 | 32.4 | - | 20.4 |
| 11 | 20.7 | 19.3 | 16.5 | 24.1 | 22.1 | 21.3 |
| 12 | 19.6 | 12.8 | 13.8 | 22.4 | 19.0 | 20.5 |
| 13 | 19.2 | 17.1 | 18.9 | 24.6 | 15.9 | 16.8 |
| 14 | 19.3 | 20.1 | 18.8 | 31.0 | 21.2 | 21.6 |
| 15 | 25.0 | 22.8 | 18.9 | 21.5 | 18.0 | 15.2 |
| 16 | 14.3 | 12.0 | 14.7 | 9.2 | 10.4 | 10.4 |
| 17 | 25.2 | 16.5 | 15.3 | 19.1 | 11.4 | 12.8 |
| 18 | 27.3 | 16.3 | 16.6 | 21.2 | 17.5 | 18.8 |
| 19 | 22.6 | 15.1 | 13.9 | 15.6 | 11.3 | 9.2 |
| 20 | 36.0 | 18.6 | 20.4 | 33.1 | 23.7 | 23.4 |
| 21 | 24.0 | 21.4 | 18.1 | 36.5 | 21.8 | 21.0 |
| 22 | 13.9 | 13.1 | 12.9 | 11.1 | 9.7 | 10.2 |
| 23 | 19.3 | 12.4 | 13.8 | 22.7 | 12.8 | 13.1 |
| 24 | 15.0 | 17.1 | 14.5 | 18.3 | 16.4 | 13.2 |
| 25 | 16.7 | 17.3 | 15.6 | 13.4 | 20.1 | 21.6 |
| 26 | 19.0 | 16.0 | 14.2 | 14.9 | 11.0 | 10.6 |
| 27 | 12.9 | 14.1 | 14.0 | 11.7 | 9.1 | 8.8 |
| 28 | 14.7 | 15.6 | 15.7 | 14.1 | 14.1 | 13.2 |
| 29 | 17.1 | 15.2 | 16.4 | 16.0 | 12.3 | 17.5 |
| 30 | 27.2 | 13.3 | 14.8 | 29.5 | 12.7 | 10.5 |
| 31 | 16.9 | 15.5 | 13.8 | 12.8 | 19.3 | 18.8 |

**Supplementary Table S1.** Individual tissue Na^+^ data (muscle and skin) of all 31 CKD patients pre renal transplantation, 3 and 6 months post renal transplantation. a.u., arbitrary units.

**Supplementary Table S2**

|  | **Na^+^ Muscle (a.u.)** | | | **Na^+^ Skin (a.u.)** | | |
| --- | --- | --- | --- | --- | --- | --- |
|  | **CKD** | **3 months** | **6months** | **CKD** | **3 months** | **6 months** |
| AR (n = 6) | 23.0 ± 3.3 | 17.0 ± 3.3 | 17.0 ± 3.2 | 23.3 ± 6.2 | 15.3 ± 5.1 | 15.6 ± 5.0 |
| No AR (n = 25) | 20.2 ± 5.3 | 16.8 ± 3.1 | 16.7 ± 2.8 | 21.0 ± 8.0 | 16.8±4.7 | 17.1 ± 5.3 |
|  |  |  |  |  |  |  |
| D.m. (n = 6) | 20.8 ± 2.8 | 16.3 ± 3.0 | 17.9 ± 3.6 | 26.1 ± 5.4 | 17.7 ± 4.7 | 18.4 ± 4.0 |
| No D.m. (n = 25) | 20.7 ± 5.5 | 16.9 ± 3.1 | 16.5 ± 2.7 | 20.3 ± 7.8 | 16.3 ± 4.8 | 16.4 ± 5.4 |
|  |  |  |  |  |  |  |
| HD (n = 3) | 24.4 ± 2.4 | 14.5 ± 1.0 | 15.9 ± 2.4 | 24.2 ± 7.5 | 12.2 ± 0.8 | 11.3 ± 3.6 |
| Preemptive RT (n = 28) | 20.3 ± 5.1 | 17.1 ± 3.1 | 16.9 ± 2.9 | 21.1 ± 7.8 | 17.0 ± 4.7 | 17.4 ± 5.1 |

**Supplementary Table S2.** Tissue Na^+^ data of various CKD sub-groups. HD group: statistical inference not feasible due to low numbers. No significant differences of tissue Na^+^ between patients with AR and without AR as well as patients with D.m. and without D.m.

AR, acute allograft rejection; D.m., Diabetes mellitus; HD, hemodialysis; RT, renal transplantation; a.u., arbitrary units.
